# Comparative Study of Different Algorithms for Human Motion Direction Prediction Based on Multimodal Data

**DOI:** 10.3390/s26020501

**Published:** 2026-01-12

**Authors:** Hongyu Zhao, Yichi Zhang, Yongtao Chen, Hongkai Zhao, Zhuoran Jiang, Mingwei Cao, Haiqing Yang, Yuhang Ding, Peng Li

**Affiliations:** 1 Key Laboratory of Intelligent Control and Optimization for Industrial Equipment of Ministry of Education, Dalian University of Technology, Dalian 116024, China; zhaohy@dlut.edu.cn; 2 School of Control Science and Engineering, Dalian University of Technology, Dalian 116024, China; 20211071041@mail.dlut.edu.cn (Y.Z.); chenyongtao@mail.dlut.edu.cn (Y.C.); jiangzr@mail.dlut.edu.cn (Z.J.); caomingwei1213@163.com (M.C.); 3 Department of Joint and Sports Medicine, The Second Hospital of Dalian Medical University, Dalian 116023, China; yanghq@dmu.edu.cn (H.Y.); dingyh@dmu.edu.cn (Y.D.)

**Keywords:** plantar pressure, motion direction prediction, CNN-BiLSTM, spatiotemporal modeling

## Abstract

The accurate prediction of human movement direction plays a crucial role in fields such as rehabilitation monitoring, sports science, and intelligent military systems. Based on plantar pressure and inertial sensor data, this study developed a hybrid deep learning model integrating a Convolutional Neural Network (CNN) and a Bidirectional Long Short-Term Memory (BiLSTM) network to enable joint spatiotemporal feature learning. Systematic comparative experiments involving four distinct deep learning models—CNN, BiLSTM, CNN-LSTM, and CNN-BiLSTM—were conducted to evaluate their convergence performance and prediction accuracy comprehensively. Results show that the CNN-BiLSTM model outperforms the other three models, achieving the lowest RMSE (0.26) and MAE (0.14) on the test set, with an R^2^ of 0.86, which indicates superior fitting accuracy and generalization ability. The superior performance of the CNN-BiLSTM model is attributed to its ability to effectively capture local spatial features via CNN and model bidirectional temporal dependencies via BiLSTM, thus demonstrating strong adaptability for complex motion scenarios. This work focuses on the optimization and comparison of deep learning algorithms for spatiotemporal feature extraction, providing a reliable framework for real-time human motion prediction and offering potential applications in intelligent gait analysis, wearable monitoring, and adaptive human–machine interaction.

## 1. Introduction

Deep learning (DL) architectures have demonstrated powerful capabilities in processing spatiotemporal data, with different models tailored to specific feature learning needs. Convolutional Neural Networks (CNNs) are effective in extracting local spatial features through convolution and pooling operations, making them suitable for capturing spatial patterns in sensor data [1]. Recurrent Neural Networks (RNNs) and their variants, such as Long Short-Term Memory (LSTM) and Bidirectional LSTM (BiLSTM), are designed to model temporal dependencies, with BiLSTM capable of capturing both past and future contextual information [2]. Hybrid models combining CNN and LSTM/BiLSTM integrate spatial and temporal feature learning, which is particularly suitable for motion direction prediction (MDP) tasks that require simultaneous analysis of spatial patterns and temporal dynamics.

Existing motion direction prediction methods can be divided into single-modal and multi-modal approaches. Single-modal methods based on visual sensors [3] are limited by environmental factors such as lighting and occlusion, while those relying on inertial sensors [4] suffer from drift errors during long-term use. Traditional deep learning models like standalone CNNs [5] excel at spatial feature extraction but fail to capture temporal dependencies, and single BiLSTM networks [6] focus on temporal modeling but lack effective spatial feature representation. Multi-modal fusion methods have emerged in recent years, but most adopt simple feature concatenation [2], which cannot fully exploit the complementary information between different data modalities.

In contrast, traditional non-DL methods for human motion prediction also exhibit clear limitations. Approaches relying solely on visual or inertial sensors [3,4] are restricted by their inherent shortcomings: visual sensors are highly sensitive to complex lighting and occlusion, reducing detection accuracy, while inertial sensors suffer from integral drift errors, resulting in cumulative prediction deviations during long-term use [3]. Moreover, at the data collection level, traditional methods typically rely on single-dimensional data collection, which limits the extraction of multi-dimensional information on human motion and leads to insufficient accuracy and robustness in motion direction prediction. Pose estimation [7] based on two-dimensional data is prone to environmental interference due to the lack of depth information. Although motion capture technology [8] can provide high-precision motion parameters, optical system-based methods are still limited by low deployment flexibility and poor adaptability in complex environments, unlike inertial measurement units (IMUs) that are portable and flexible [1,9].

In recent years, advances in motion capture technology [10,11] have enabled the creation of large-scale human motion datasets, and deep learning-based prediction algorithms have proliferated; however, motion analysis that incorporates the prediction of individual motion direction in both civilian and military scenarios remains a significant challenge. Currently, most human motion datasets are acquired using optical motion capture systems [12], which are captured in controlled laboratory environments and processed extensively to form structured datasets, leading to the primary data source for MDP studies being overly reliant on optical motion capture technology [13] while ignoring the potential of plantar pressure data—an interface between the human body and the external environment with strong correlation to motion states and stress responses. Additionally, existing multi-modal fusion methods for MDP fail to fully exploit cross-modal complementary information, and there is a lack of targeted research integrating plantar pressure [14] and IMU data [15] to meet the dual demands of civilian and military motion analysis.

Driven by advances in artificial intelligence and its deep integration with biomechanics, research on human motion has proven essential for both civilian and military applications, with motion direction prediction (MDP) [16] being a critical link in motion analysis, enabling proactive interventions and performance enhancement by anticipating future motions. With the rapid aging of the global population, the World Health Organization (WHO) estimates that by 2050, individuals aged 60 and above will account for 22% of the total population [17]. Civilians demand efficient and accurate MDP technologies for intelligent rehabilitation [18] and human–computer interaction [19] amid global aging, while the military requires real-time MDP for soldier state assessment, combat effectiveness maintenance, and operational decision support [20]; emerging industries like smart wearable devices [21] and autonomous driving [22,23] also raise high demands for real-time and reliable MDP.

Against this backdrop, this study, grounded in practical application requirements, integrates multi-dimensional plantar pressure data [24,25] with inertial measurement unit (IMU) data. Results of our preliminary experiments demonstrate that single-modal models not only yield significantly lower prediction accuracy than multimodal fusion models, but also exhibit marked performance fluctuations across different subject groups. To address these issues, this study proposes a multimodal fusion framework that integrates plantar pressure and IMU data and develops a hybrid CNN-BiLSTM model to achieve joint spatiotemporal feature learning. This constitutes the core novelty of the present study: by taking full advantage of convolutional neural networks (CNNs) in spatial feature extraction and bidirectional long short-term memory networks (BiLSTMs) in modeling bidirectional temporal dependencies, the proposed model overcomes the inherent limitations of both single-modal models and simple hybrid models.

The remaining structure of this paper is arranged as follows: Section II describes the multimodal data acquisition system and experimental procedures used to construct the dataset and presents four different prediction models. Section III analyzes the col-lected plantar pressure and inertial data to extract representative motion features and compares four different prediction models experimental results, and discusses model performance. Finally, Section IV summarizes the conclusions and outlines directions for future research.

## 2. Human Motion Acquisition Data Experiment and Comparison of Prediction Models

This section elaborates on the human motion data acquisition experiment, covering the data acquisition system, experimental procedures, and data processing links. This study includes hardware selection, experimental design, and dataset construction, thereby establishing a robust experimental framework to support the training and validation of subsequent algorithmic models, and will also introduce the adopted deep learning models and corresponding evaluation metrics.

### 2.1. Data Acquisition System

**Figure 1:**
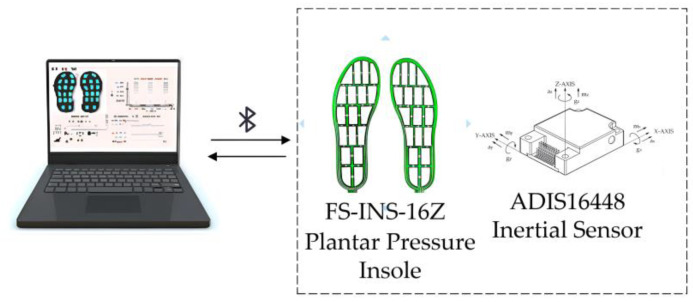
Overall Design of the Multi-modal Data Acquisition System.

This study developed a multi-modal data acquisition system for capturing both plantar pressure and inertial sensor data, which primarily comprises plantar pressure and inertial sensors. The overall system architecture is illustrated in Figure 1.

#### 2.1.1. Plantar Pressure Insole

**Figure 2:**
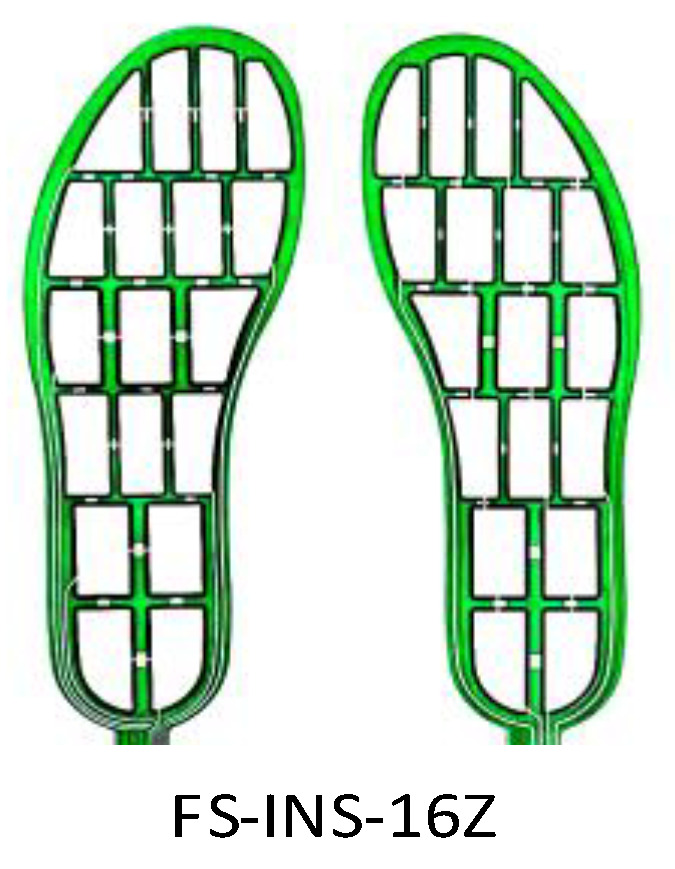
FS-INS-16 Plantar Pressure Insole.

The FS-INS-16 plantar pressure insole [26], as shown in Figure 2, comprises 16 independent sensing regions for accurate plantar pressure measurement.

Plantar pressure signal acquisition adopts the FS-INS-16 flexible thin-film pressure sensor. Based on the piezoresistive sensing principle, this sensor features flexible fitting characteristics, which can adapt to different foot shapes and is suitable for pressure monitoring under dynamic gait. When plantar pressure is applied, the internal resistance of the sensing area changes dynamically and is converted into corresponding voltage signals to achieve precise data acquisition. This design enables the insole to accurately capture the pressure distribution characteristics and temporal variation patterns of different foot regions during movements such as walking, running, and turning: in the temporal dimension, it records pressure fluctuations synchronized with gait rhythms; in the spatial dimension, it presents pressure distribution differences in various regions such as the arch and heel. The obtained high-precision and multi-dimensional data provide key basic information for analyzing human motion intentions and predicting motion directions. The sensor integrates 16 independent sensing units, which are precisely arranged according to the anatomical and mechanical divisions of the human plantar, enabling synchronous acquisition of pressure signals in key plantar regions. All sensing units have a unified acquisition parameter of instantaneous vertical pressure, with a measuring range of 0.5–10 kg, a measurement accuracy of ±15%, and a response time of less than 10 μs, which can meet the real-time requirements of dynamic gait acquisition. The high-precision and multi-dimensional data obtained thereby provide a key basis for analyzing human motion intentions and predicting motion directions.

#### 2.1.2. Inertial Sensor

**Figure 3:**
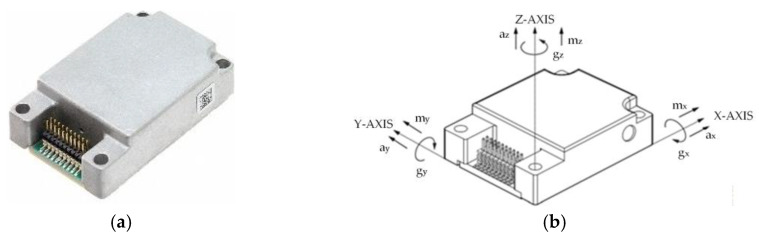
(**a**) ADIS16448 Inertial Sensor product image; (**b**) ADIS16448 Inertial Sensor direction Reference.

The ADIS16448 inertial sensor [27], as shown in Figure 3, integrates a three-axis gyroscope, a three-axis accelerometer, and a three-axis magnetometer, enabling comprehensive acquisition of dynamic motion information.

In this study, only the accelerometer module is utilized. It independently records linear acceleration along the X, Y, and Z axes, thereby capturing dynamic variations in the anterior–posterior, medial-lateral, and vertical directions of human motion. These measurements characterize the multi-dimensional dynamics of motion and contribute to the interpretation of motion intentions. When combined with plantar pressure data, they provide complementary information that enhances the analysis of pressure distribution and overall motion patterns.

All collected plantar pressure and inertial data are transmitted to a personal computer via Bluetooth (as shown in Figure 1), forming a reliable basis for motion analysis and Motion direction prediction.

### 2.2. Experiment Procedure

To ensure the quality of data collection in the human motion experiment, this study recruited 6 healthy volunteers with an average height of approximately 1.75 m and an average weight of about 65 kg. All participants were young males aged 20–23 years, with physical characteristics within the normal range of the general population, which ensures that the collected data are both representative and widely applicable. By minimizing the impact of individual physiological differences, this experimental design enhances the reliability and generalizability of the experimental results. First, this study adopted a continuous motion capture approach combined with post hoc data segmentation based on sensor signal characteristics. Second, each subject completed 10 repetitions of the walking task. To avoid fatigue-induced motion deviations, we arranged a 10 min rest interval between repetitions to ensure that subjects fully recovered before proceeding to the next test.

**Figure 4:**
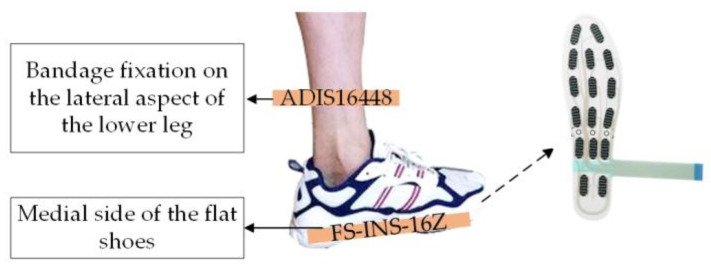
Schematic Diagram of Sensor Installation.

The data collection experiment was conducted on a flat walking surface. As illustrated in Figure 4, sensors were attached to both feet of the participants to ensure optimal signal acquisition. The plantar pressure sensors sampled data at 100 Hz, while the inertial sensors operated at 400 Hz. Each participant performed the walking task continuously for approximately 40 s.

During data acquisition, the data acquisition system continuously records plantar pressure and inertial signals in real time, allowing for the precise capture of subtle variations in human motion.

### 2.3. Data Processing

The collected data were processed using low-pass filtering, down-sampling, and related preprocessing procedures to improve data quality and construct the final dataset.

#### 2.3.1. Low-Pass Filtering

During data acquisition, the recorded signals are often contaminated by high-frequency noise sources, such as electronic interference and motion artifacts.

Since the effective information of gait and plantar pressure variations primarily lies within the low-frequency range, the raw data are processed using a fourth-order Butterworth low-pass filter. This filter is chosen for its maximally flat magnitude response within the passband and its monotonic attenuation at higher frequencies. The corresponding frequency response is given by:

(1) $${\left|H\left(j\omega \right)\right|}^{2}\;=\;\frac{1}{1+{\left(\frac{\omega }{\omega_{c}}\right)}^{2n}}$$

where *n* denotes the filter order that determines the steepness of the transition band; *ω* represents the angular frequency; and *ω_c_* is the cutoff angular frequency.

In discrete-time systems, the analog filter must be transformed into its digital equivalent, which is achieved using the bilinear transform method:

(2) $$s=\frac{2}{T_{s}}\frac{1-z^{-1}}{1+z^{-1}}$$

where *T_s_* denotes the sampling interval.

To prevent phase distortion during filtering, a zero-phase filtering approach is employed. The input sequence is first filtered in the forward direction to produce an intermediate output, which is then reversed and filtered backward. A final reversal of this signal yields the zero-phase filtered result. After low-pass filtering, frequency components above 5 Hz are effectively attenuated, preserving the primary characteristics of the motion signals.

#### 2.3.2. Data Synchronization

As the inertial data are sampled at 400 Hz and the plantar pressure data at 100 Hz, the inertial signals are down-sampled to ensure consistent data length across modalities. Resampling is performed using a frequency-domain approach, in which the sampling rate is adjusted through Fourier transform and interpolation.

Let the original signal be $x\left[n\right]\;\left(n=0,1,\ldots ,N-1\right)$ with a sampling frequency of $f_{s}=400\;Hz$. Its discrete Fourier transform (DFT) is given by:

(3) $$X\left[k\right]=\sum_{n=0}^{N-1}x\left[n\right]e^{-j2\pi kn/N},k=0,1,\ldots ,N-1$$

where $X\left[k\right]$ represents the spectral components of the signal at frequency $f=kf_{s}/N$, providing its frequency-domain representation.

For the target sampling rate of 100 Hz, the corresponding Nyquist frequency is $f_{Nyq}^{\prime }=50\;Hz$. To suppress aliasing, only the spectral components within this frequency range are retained. Given a new number of sampling points M, the truncated spectrum is expressed as:

(4) $$X^{\prime }\left[k\right]=\left\{\begin{array}{ll} X\left[k\right], & 0\leq k\leq M/2\\ X\left[N-M/2+k\right], & M/2\leq k<N\\ 0, & otherwise \end{array}\right.$$

where *N* and *M* denote the original and resampled sequence lengths, respectively. This operation preserves the low-frequency components while discarding high-frequency content that could lead to aliasing during resampling.

The down-sampled time-domain signal is then reconstructed using the inverse discrete Fourier transform (IDFT), expressed as:

(5) $$x^{\prime }\left[m\right]=\frac{1}{M}\sum_{k=0}^{M-1}X^{\prime }\left[k\right]e^{j2\pi km/M},m=0,1,\ldots ,M-1$$

The resulting signal $x^{\prime }\left[m\right]\;$ represents the down-sampled version of the original inertial data, corresponding to an equivalent sampling rate of 100 Hz.

#### 2.3.3. Dataset Creation

First, data reading and cleaning were performed. The dataset comprises 32 feature columns and one target column. The values in the 33rd column, containing semicolon-separated entries, were converted into floating-point numbers, and all rows with missing values were removed. Missing values in the dataset were mainly caused by temporary signal interference during Bluetooth data transmission and occasional sensor contact instability. After cleaning, the remaining valid data accounted for 98.7% of the original data, which is sufficient to support subsequent model training and validation. Subsequently, both the feature and target variables were normalized to the range [0, 1] through Min–Max normalization. This step eliminates the influence of inconsistent units (i.e., dimensional differences) and accelerates the convergence of the prediction algorithm. The normalization process is defined as:

(6) $$x^{\prime }=\frac{x-\min\left(D\right)}{\max\left(D\right)-\min\left(D\right)}$$

where *D* denotes the original dataset, *x* represents an individual data point, and $x^{\prime }$ is the normalized value. The numerator $\left(x-\min\left(D\right)\right)$ shifts the data so that its minimum maps to zero, while the denominator $\left(\max\left(D\right)-\min\left(D\right)\right)$ scales the values into the interval $\left[0,1\right]$.

After normalization, a time-series dataset was constructed for one-step-ahead prediction. Specifically, the previous 10-time steps were used as input to forecast the value at the next time step. A sliding-window function was applied to generate the input samples and their corresponding target values. Finally, the dataset was divided into training and test sets in an 8:2 ratio, forming the foundation for subsequent motion direction prediction.

### 2.4. Motion Parameter Prediction Model

To comprehensively evaluate the influence of different neural network architectures on motion prediction, four deep learning models are implemented and compared: a Convolutional Neural Network (CNN), a Bidirectional Long Short-Term Memory (BiLSTM) network, a hybrid CNN-LSTM model, and a hybrid CNN-BiLSTM model. Each model is designed to extract and integrate spatial and temporal features from multi-source sensor data. The core structural configurations and functional principles of these models are detailed below.

#### 2.4.1. CNN Prediction Algorithm

In the initial convolutional layer, the algorithm captures local dynamic features of the time-series data using 64 convolution kernels, each processing signal across three consecutive time steps. The subsequent pooling layer halves the temporal dimension to preserve the most salient features. In the second convolutional layer, 32 convolution kernels further refine high-level representations by scanning two-time steps each, enabling the transition from basic signal characteristics to abstract motion patterns. The fully connected layer then flattens the three-dimensional feature map into a one-dimensional vector, where nonlinear feature relationships are combined and mapped. During training, a dropout rate of 30% is applied to mitigate overfitting. Finally, a regression layer outputs the predicted motion direction [5,28].

#### 2.4.2. BiLSTM Prediction Algorithm

The BiLSTM prediction algorithm [6,29] employs a bidirectional structure, taking 32-dimensional input data over ten-time steps. The forward LSTM processes the sequence from t = 1 to t = 10 to capture historical dependencies, while the backward LSTM processes from t = 10 to t = 1 to capture future contextual information. The outputs from both directions are concatenated into a 200-dimensional vector, with 60 neurons randomly dropped to prevent overfitting. At the final time step, the concatenated output is reduced to 100 dimensions, and 30 neurons are randomly masked before generating the final prediction output.

#### 2.4.3. CNN-LSTM Prediction Algorithm

The CNN-LSTM hybrid prediction algorithm [2,30] integrates convolutional and recurrent layers to combine spatial and temporal feature extraction. The front-end comprises two convolutional layers and one pooling layer for feature preprocessing. The first convolutional layer, containing 64 kernels with a stride of 3, focuses on extracting short-term motion features. The pooling layer compresses the temporal dimension to five-time steps, effectively reducing data dimensionality. The second convolutional layer, composed of 32 kernels with a stride of 2, performs further refined feature extraction before passing the features to the LSTM module for temporal modeling.

#### 2.4.4. CNN-BiLSTM Prediction Algorithm

The CNN-BiLSTM prediction algorithm extends the CNN-LSTM structure by incorporating a bidirectional recurrent mechanism. Two convolutional layers and one pooling layer precede the BiLSTM module, configured identically to the CNN-LSTM model for consistent feature extraction. A dropout rate of 30% is applied in the CNN module and 40% in the BiLSTM module, as BiLSTM networks are more prone to over-fitting. Compared with the CNN-LSTM model, the bidirectional mechanism enhances the model’s ability to analyze both preceding and succeeding motion dependencies, thereby improving the interpretability of temporal relationships.

**Figure 5:**
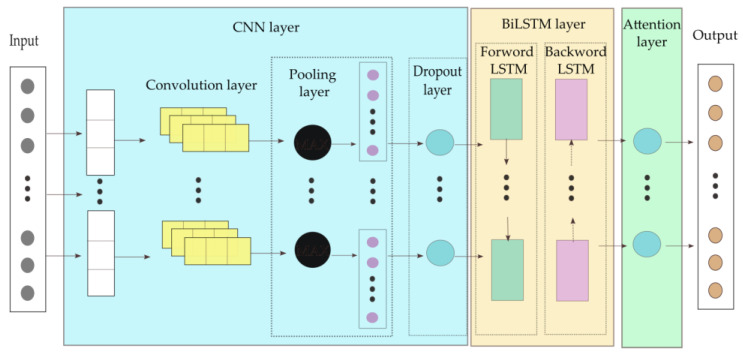
CNN-BiLSTM Structure.

Through comparative analysis of the above four models, the CNN-BiLSTM architecture effectively integrates the advantages of CNN and BiLSTM, accurately adapting to the processing needs of motion-related time-series data. Meanwhile, the CNN module in this architecture has the exact same configuration as that in the CNN-LSTM model. To avoid redundancy, only the overall architecture of the CNN-BiLSTM model is presented here, as shown in Figure 5.

### 2.5. Algorithm Evaluation Metrics

In the human motion direction prediction task, three evaluation metrics are employed to comprehensively assess model performance: Root Mean Square Error (RMSE), Mean Absolute Error (MAE), and the Coefficient of Determination (R^2^). RMSE quantifies the deviation between predicted and actual values, reflecting the stability of model predictions. MAE measures the average absolute difference between predicted and actual values, providing intuitive interpretability. R^2^ evaluates the model’s goodness of fit by quantifying its ability to explain the variance in target variables.

Together, these metrics enable a comprehensive evaluation of prediction accuracy, robustness, and explanatory power. The corresponding formulas are as follows:

(7) $$RMSE=\sqrt{\frac{1}{n}\sum_{i=1}^{n}{\left(y_{i}-\hat{y_{i}}\right)}^{2}}$$

(8) $$MAE=\frac{1}{n}\sum_{i=1}^{n}\left|y_{i}-\hat{y_{i}}\right|$$

(9) $$R^{2}=1-\frac{\sum_{i=1}^{n}{\left(\hat{y_{i}}-y_{i}\right)}^{2}}{\sum_{i=1}^{n}{\left(\bar{y_{i}}-y_{i}\right)}^{2}}$$

where $y_{i}$ is the actual value, $\hat{y_{i}}$ is the predicted value, $\bar{y_{i}}$ is the mean of the actual values, and *n* is the number of samples.

## 3. Results and Discussion

This section analyzes the preprocessed data to extract representative motion features: plantar pressure data describes the distribution and variation patterns of ground reaction forces, while inertial data captures changes in human body acceleration. Integrating these two modal data enables comprehensive characterization of motion states, providing support for accurate motion recognition and direction prediction. Additionally, quantitative evaluation metrics are used to conduct a comprehensive performance comparison and analysis of multiple deep learning models; by evaluating the predictive performance of each model, the optimal algorithm suitable for motion prediction is subsequently identified.

### 3.1. Analysis of Plantar Pressure Data

As a key technique for interpreting foot function and gait characteristics, plantar pressure distribution analysis plays an important role in medical diagnosis, sports science, and product design. Dynamically, normal plantar pressure progresses sequentially from the heel to the arch, metatarsal, and toe during a complete gait cycle, which consists of the stance and swing phases. In the stance phase, the gradual shift of pressure from the heel to the forefoot reflects real-time regulation of the body’s center of gravity—for instance, an excessive medial shift increases pressure on the inner plantar region. During the swing phase, pressure variations are closely associated with the coordinated motion of the hip, knee, and ankle joints. Their range, speed, and synchronization determine the contact area and duration between the sole and the ground, shaping the characteristic pressure distribution of this phase.

**Figure 6:**
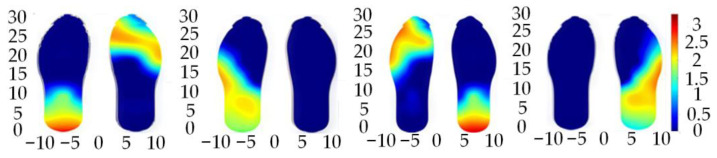
Plantar Pressure Heatmap During Walking.

In this study, plantar pressure data while walking are analyzed using a heatmap visualization approach, as illustrated in Figure 6.

The heat maps reveal clear dynamic patterns in plantar pressure throughout the gait cycle. Taking the left foot as an example, the sequence of heatmaps from left to right illustrates plantar pressure progression during a gait cycle, from heel contact to full-foot support and forefoot push-off.

In the heatmaps, regions closer to red indicate higher pressure, whereas regions closer to blue indicate lower pressure. Across walking phases, pressure is primarily concentrated in the heel and forefoot, with relatively low loading on the arch, consistent with normal gait mechanics: heel initially contacts the ground to bear body weight, after which the center of gravity shifts forward, and the forefoot generates force for push-off. Comparison between the left and right feet shows similar pressure distribution and intensity, indicating bilateral symmetry and the alternating support of both feet to maintain balance and stability during walking.

**Figure 7:**
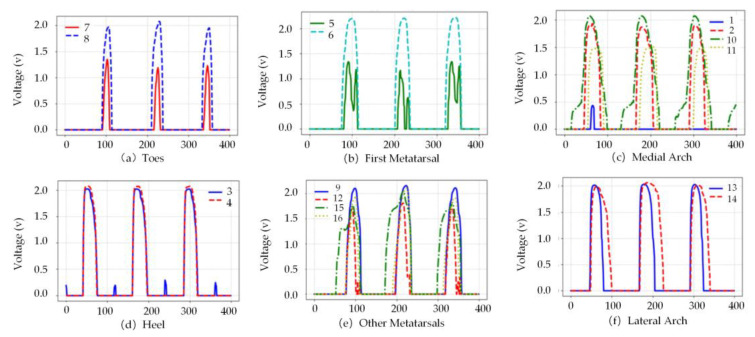
Pressure profiles of different plantar regions.

This study also presents regional plantar pressure curves during walking, as shown in Figure 7. The experimental data indicate that the dynamic pressure patterns in each region correspond closely with the distribution observed in the heatmaps. These results complement the static heatmap, collectively illustrating the dynamic characteristics of plantar pressure distribution.

### 3.2. Analysis of Acceleration Data

**Figure 8:**
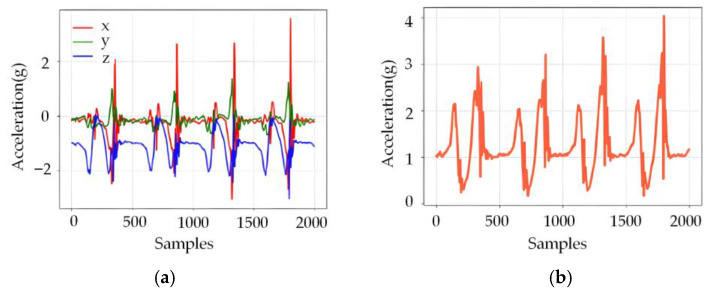
Acceleration Curve During Walking, (**a**) X/Y/Z-axis acceleration; (**b**) Resultant acceleration.

To further investigate human motion dynamics, triaxial acceleration data of the subjects’ feet were collected under various motion conditions using an Inertial Measurement Unit (IMU) ADIS16448. As an example, Figure 8 illustrates the variations in each acceleration axis and the resultant acceleration during walking.

In the left Figure 8, the curves of different colors represent the acceleration variations along the x-, y-, and z-axes, respectively. The three curves exhibit clear periodic fluctuations corresponding to the cyclic motion of the foot during walking. Larger acceleration changes occur during heel contact, with corresponding responses during forefoot push-off, reflecting the alternating motion phases of the gait cycle.

In the right Figure 8, the red curve shows the resultant acceleration, which also presents distinct periodic fluctuations. Its fluctuation period closely matches that of the triaxial acceleration curves, further confirming the periodic nature of walking motion.

Overall, the acceleration signals display consistent regularity in peak patterns and amplitudes. These characteristics, together with plantar pressure features, provide a significant basis for predicting human motion direction.

### 3.3. Analysis of Experimental Results

To validate the effectiveness of the motion prediction models, this section presents a comprehensive analysis of their experimental results. The evaluation focuses on model convergence behavior, prediction accuracy, and generalization performance. By comparing the loss curves and key quantitative indicators of four different neural network architectures—CNN, BiLSTM, CNN-LSTM, and CNN-BiLSTM—the optimal model structure and its advantages in spatial–temporal feature learning and motion direction prediction are identified.

**Figure 9:**
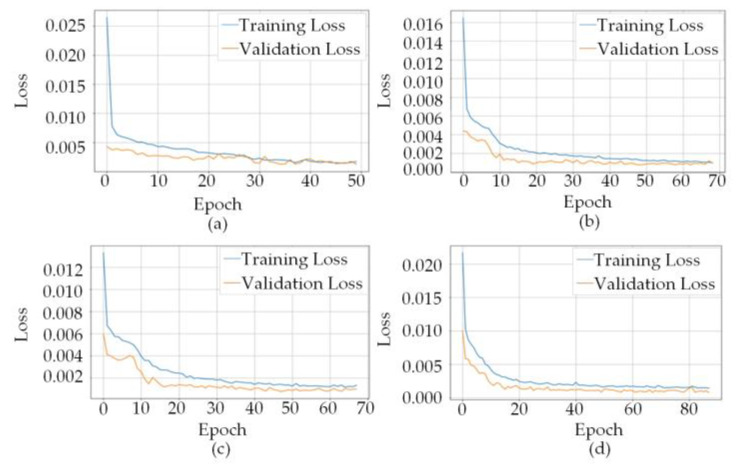
(**a**) Loss Curve of CNN Prediction Algorithm; (**b**) Loss Curve of BiLSTM Prediction Algorithm; (**c**) Loss Curve of CNN-LSTM Prediction Algorithm; (**d**) Loss Curve of CNN-BiLSTM Prediction Algorithm.

By training the dataset using different prediction algorithms, the corresponding loss curves were obtained, as illustrated in Figure 9. In these figures, the blue curves indicate the training loss, whereas the orange curves represent the validation loss.

### 3.4. Model Convergence and Generalization Analysis

A comparative summary of the convergence performance of the four algorithms is presented in Table 1.

As shown in Figure 9 and Table 1, the algorithms exhibit clear differences in convergence behavior and generalization capability. The CNN and BiLSTM algorithms converged rapidly but exhibited relatively high final losses (0.001–0.002), while the CNN-LSTM model required more epochs, achieving stable but suboptimal validation accuracy (training loss 0.02, validation loss 0.04). In contrast, the CNN-BiLSTM algorithm, despite the longest training duration, attained an exceptionally low final loss near 0.000, with rapid initial convergence followed by steady refinement, indicating both efficient feature capture and detailed motion learning.

Regarding overfitting suppression and generalization, the CNN algorithm achieves limited generalization through regularization, while BiLSTM mitigates overfitting via bidirectional temporal modeling but lacks robust spatial representation. CNN-LSTM improves stability with temporal regularization and data augmentation, yet a small gap between training and validation loss persists. In contrast, the CNN-BiLSTM model combines BiLSTM’s overfitting suppression with CNN’s spatial feature extraction, maintaining consistently low validation loss and a minimal generalization gap, thereby demonstrating superior adaptability and comprehensive generalization performance.

### 3.5. Model Prediction Performance Evaluation

To further assess predictive performance, three metrics—RMSE, MAE, and R^2^—were calculated on both the training and test sets. The results are summarized in Table 2.

As shown in Table 2, the CNN model performs well on the training set (RMSE = 0.20, MAE = 0.12, R^2^ = 0.86), but its test R^2^ drops to 0.48, indicating overfitting. The BiLSTM model yields similar test performance yet exhibits higher training error, suggesting limited spatial feature representation. The CNN-LSTM model performs the worst overall, with the lowest R^2^ (0.16 on test set), reflecting inadequate coordination in its hybrid architecture.

In contrast, the CNN-BiLSTM model achieves the best overall performance. Both its training and test errors (RMSE = 0.19/0.26; MAE = 0.11/0.14) are the lowest among all models, while its R^2^ values (0.90 train/0.86 test) indicate excellent fitting accuracy and generalization. The small R^2^ gap (0.04) between training and test sets demonstrates strong robustness and minimal overfitting.

This superior performance arises from the effective extraction of spatial local features by the CNN module and the precise capture of bidirectional temporal dependencies by the BiLSTM module. Together, these enable spatial-temporal joint modeling, resolving the long-standing challenge of balancing fitting accuracy and generalization. Consequently, the CNN-BiLSTM algorithm is confirmed to be the most suitable and robust model for human motion direction prediction.

### 3.6. Discussion

In terms of feature extraction and model adaptability, the CNN algorithm relies on local convolutional perception and weight sharing to extract spatial features, but it lacks the ability to capture dynamic motion information. The BiLSTM algorithm emphasizes temporal dependencies but provides limited spatial representation, reducing its adaptability to multimodal data. The CNN-LSTM model combines spatial and temporal feature extraction; however, its unidirectional temporal modeling restricts causal motion analysis and overall architectural synergy.

By contrast, the CNN-BiLSTM model realizes a more advanced spatial–temporal joint modeling strategy. The CNN layers effectively extract spatial representations of plantar pressure, while the BiLSTM layers capture bidirectional temporal dependencies, enabling the model to learn both dynamic motion transitions and spatial correlations. This hybrid architecture exhibits highly consistent convergence paths and synchronous low-loss behavior, resulting in markedly enhanced stability and adaptability in human motion direction prediction compared with the other algorithms.

In summary, compared with the CNN, BiLSTM, and CNN-LSTM algorithms, the CNN-BiLSTM model demonstrates more effective training cycle planning, enhanced overfitting suppression, and superior spatial-temporal feature fusion. It exhibits comprehensive advantages reflected in its lower loss value, more stable convergence trajectory, and stronger generalization capability. Overall, the CNN-BiLSTM algorithm proves to be the most suitable and robust model for human motion direction prediction among the four evaluated approaches.

## 4. Conclusions and Future Work

We tested four neural network models for predicting human motion direction: CNN, BiLSTM, CNN-LSTM, and CNN-BiLSTM. The CNN-BiLSTM model stood out in terms of how quickly it converged, how accurate its predictions were, and how well it generalized to new data. Its combined structure lets it learn both spatial and temporal features at the same time—this meant it had low loss, stabilized quickly during training, and held up well even in variable conditions. By using CNN layers to pull out spatial details and BiLSTM layers to model temporal patterns in both directions, the CNN-BiLSTM model kept loss low, trained steadily, and performed reliably on both training and test data. All in all, this model gives us a solid base for smart motion prediction, and it could work well for things like gait analysis, tracking sports performance, or helping with rehabilitation.

That said, the current CNN-BiLSTM model is not perfect. Right now, we just stack features together when fusing them, which might waste information or miss key details when motion changes fast. Also, data collection relies on Bluetooth, so it cannot be used in real time for long-distance or outdoor scenarios.

Future work will focus on enhancing model adaptability and system scalability. Potential improvements include:

- Developing a dynamic feature fusion mechanism (e.g., attention-based or cross-modal correlation learning) to strengthen interaction between spatial and temporal features;
- Expanding the dataset to cover more diverse motion patterns and scenarios, improving generalization;
- Expanding the validation set data such that the test set includes diverse data unseen by the model during training, thereby avoiding biases;
- Expanding the participant pool to include samples from more age groups, body types, and pathological gait cases;
- Conducting experiments on complex terrains, supplementing k-fold cross-validation, and performing quantitative comparisons with state-of-the-art methods;
- Integrating advanced communication technologies for real-time, long-distance data acquisition and processing.

## Figures and Tables

**Table 1:** Comparison of convergence performance of four prediction algorithms.

Algorithm	CNN	BiLSTM	CNN-LSTM	CNN-BiLSTM
Training Epochs	45	50	60	80
Training Loss	0.002	0.001	0.002	0.000
Validation Loss	0.002	0.001	0.004	0.005

**Table 2:** Comparison of model prediction performance.

Algorithm	RMSE		MAE		R^2^	
	Train	Test	Train	Test	Train	Test
CNN	0.20	0.34	0.12	0.23	0.86	0.48
Bi-LSTM	0.25	0.34	0.15	0.20	0.76	0.48
CNN-LSTM	0.35	0.44	0.25	0.32	0.53	0.16
**CNN-BiLSTM**	**0.19**	**0.26**	**0.11**	**0.14**	**0.90**	**0.86**

## Data Availability

Data are only available on request due to privacy restrictions.
